# The role of mathematical modelling in guiding the science and economics of malaria elimination

**DOI:** 10.1016/j.inhe.2010.09.005

**Published:** 2010-12

**Authors:** Richard J. Maude, Yoel Lubell, Duong Socheat, Shunmay Yeung, Sompob Saralamba, Wirichada Pongtavornpinyo, Ben S. Cooper, Arjen M. Dondorp, Nicholas J. White, Lisa J. White

**Affiliations:** a Mahidol–Oxford Tropical Medicine Research Unit, Faculty of Tropical Medicine, Mahidol University, 3/F, 60th Anniversary Chalermprakiat Building, 420/6 Rajvithi Road, Rajthevee, Bangkok 10400, Thailand; b Centre for Tropical Medicine, Nuffield Department of Clinical Medicine, CCVTM, University of Oxford, Churchill Hospital, Old Road, Oxford OX3 7LJ, UK; c Department of Infection and Tropical Medicine, Heartlands Hospital, Bordesley Green East, Birmingham B9 5SS, UK; d Cambodia National Malaria Centre, #372 Boulevard Monivong, Corner Street 322, Phnom Penh, Cambodia; e London School of Hygiene & Tropical Medicine, Keppel Street, London WC1E 7HT, UK

**Keywords:** Mathematical modelling, Model, Economic, Malaria, Policy, Control

## Abstract

Unprecedented efforts are now underway to eliminate malaria from many regions. Despite the enormous financial resources committed, if malaria elimination is perceived as failing it is likely that this funding will not be sustained. It is imperative that methods are developed to use the limited data available to design site-specific, cost-effective elimination programmes. Mathematical modelling is a way of including mechanistic understanding to use available data to make predictions. Different strategies can be evaluated much more rapidly than is possible through trial and error in the field. Mathematical modelling has great potential as a tool to guide and inform current elimination efforts. Economic modelling weighs costs against characterised effects or predicted benefits in order to determine the most cost-efficient strategy but has traditionally used static models of disease not suitable for elimination. Dynamic mathematical modelling and economic modelling techniques need to be combined to contribute most effectively to ongoing policy discussions. We review the role of modelling in previous malaria control efforts as well as the unique nature of elimination and the consequent need for its explicit modelling, and emphasise the importance of good disease surveillance. The difficulties and complexities of economic evaluation of malaria control, particularly the end stages of elimination, are discussed.

## 1 Introduction

Elimination of malaria is defined as the interruption of local mosquito-borne malaria transmission in a defined geographic area, i.e. incidence of zero contracted cases.^1^ This is distinct from eradication (worldwide removal of a malaria parasite species) and control (reducing the malaria burden so that it is no longer a public health problem).^1^ The Global Malaria Eradication Programme (GMEP) attempted to eliminate malaria from low transmission areas of the world in the 1950s and 1960s and, despite success in 37 of 143 endemic countries and large reductions in many others, it ultimately failed. The goal of malaria control was then adopted in its place.^1^ Over the past 5 years, funding and political commitment for malaria control have again increased markedly. Consequent reductions in malarial morbidity and mortality in several endemic countries as well as strong global advocacy have resulted in global eradication being considered as the new target.^1^ The WHO now aims to reduce greatly the malaria burden in high transmission areas and to eliminate malaria from low transmission areas with the ultimate goal of global malaria eradication as more robust tools becomes available.^1^

Of all the infectious diseases of humans, only smallpox has been eradicated. This disease had the advantage of a single potent control measure, i.e. a highly effective vaccine with a long duration of protection. No intervention of comparable efficacy is available for malaria and the transmission dynamics of malaria are more complex than smallpox, making it potentially more difficult to eliminate. Many malaria control tools are now available, the most potent of which are artemisinin-based combination therapies (ACT), insecticide-treated bed nets and indoor residual spraying with insecticide. However, it is likely that none of these are sufficiently effective on their own to achieve elimination, even with the possible future addition of vaccination, and various combinations of targeted control strategies are probably required.^2^, ^3^, ^4^, ^5^, ^6^ Widely varying malaria epidemiology and availability of resources mean that the ideal combination of strategies will vary between locations.^6^

It is not possible to trial all malaria control interventions in all settings, and few studies have aimed for elimination.^7^ The data on which countries can base malaria elimination policy decisions are thus usually limited and frequently non-existent.^4^ Somehow these limited data must be used to predict the effect of scale-up of interventions beyond the areas and populations in which they were originally studied. By combining these limited data with detailed mechanistic understanding, mathematical modelling is able to do this and can evaluate different strategies and the effect of confounders much more rapidly than is possible through trial and error in the field. It also allows exploration of why particular control measures may be more effective in a particular setting, thus providing the opportunity to optimise these and devise new strategies.^6^ During the GMEP, mathematical modelling assisted by clarifying the primacy of interruption of transmission by vector control, supporting widescale use of the insecticide dichlorodiphenyltrichloroethane (DDT). In the current era, we are fortunate to have more powerful means of manipulating data using computers. Mathematical modelling of malaria transmission and methods of economic evaluation are consequently much more developed. The combination of these two, together with high-quality surveillance data, has great potential as a pragmatic tool to help guide malaria elimination efforts and in particular to predict which are likely to be the most efficient and cost-effective strategies in different epidemiological settings. To date, however, they have remained largely separate disciplines.

## 2 Guiding the science

### 2.1 Modelling and malaria control policy

Application of mathematics to aid logical reasoning around interventions to reduce malaria transmission was pioneered by Ronald Ross in the early 1900s.^2^, ^8^ He argued that mathematics is a way to apply careful reasoning to a problem and from his calculations deduced that, for example, to eliminate malaria one merely had to reduce transmission below a certain threshold level and that combinations of interventions are generally more effective than single interventions.^8^, ^9^ Unfortunately at the time his work was largely ignored by those who were planning such interventions, although later his conclusions were proven to be correct.^8^, ^9^, ^10^

In the mid 1900s, George MacDonald further developed Ross's model and used it to demonstrate the importance of vector control and interruption of transmission in malaria elimination.^11^ MacDonald concluded that this would be much easier to achieve in low transmission settings. MacDonald's model did not include many features important in malaria transmission, e.g. human population dynamics, seasonal vector dynamics, superinfection and elements of the malaria life cycle.^8^ DDT was then widely used to kill mosquitoes and, together with the then still highly effective antimalarial chloroquine, had great success in reducing the malaria burden. Dwindling investment in malaria control as well as DDT resistance and concerns about its ecological impact resulted in it being generally withdrawn from use in the 1970s.^10^

In 1966, a computer simulation based on MacDonald's work was used to plan a malaria control field research trial using DDT spraying and mass drug administration (MDA) in Kankiya, Nigeria. The results differed greatly from the model predictions, with the intervention being far less effective than predicted, and it was thought this was due both to difficulty obtaining accurate parameter estimates for key epidemiological indices and an inability of these indices and the model structure to describe adequately the epidemiology of malaria in that area.^12^ Models are always simpler than reality and thus often fail to capture the degree of heterogeneity present in reality. This homogeneity usually leads to overestimation of the impact of a control measure and thus ‘exaggerated optimism’.^9^ MacDonald's model makes a number of simplifying assumptions, including homogeneous transmission in an area, no acquired immunity and mosquitoes biting randomly.^9^ It is not sufficiently realistic to be suitable for detailed design of control strategies at the implementation level, although its conclusions have proven very useful conceptually.^13^

A few years later, another model was developed as an integral part of the Garki Project in Nigeria (1969–1976). This model included the addition of human immunity to a more sophisticated derivative of MacDonald's model. It was used to explore the impact of vectorial capacity on the incidence and prevalence of malaria infection in humans and aimed to predict the effects of specific control measures (larvicide, adulticide and MDA), alone and in combination.^14^ Although it achieved its first aim reasonably well, it was unable to reproduce accurately the effect of control measures. Although a significant advance from previous models, the Garki model suffered from oversimplifying assumptions and difficulties with quantifying accurately many of the input parameters. Ongoing model development was limited to testing the baseline model against new data and subsequent adjustment of only the vectorial capacity, without changing much of the model structure and most of the parameters. The Garki model helped increase the rigour of study design but, owing to its limitations, remained primarily a teaching tool.

**Figure 1:**
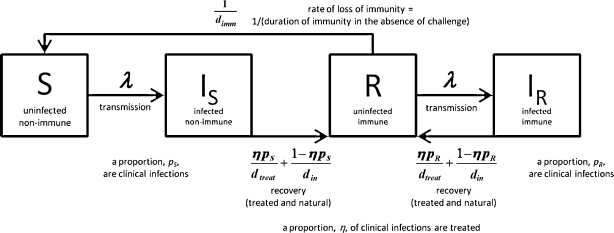
A simple deterministic mathematical model of malaria transmission. The diagram shows the compartmental structure of the model with time-dependent variables: *S*, uninfected and non-immune; *I*_S_, infected with no prior immunity; *R*, uninfected with immunity; *I*_R_, infected with prior immunity; *d*_*treat*_, average duration of treated infection; *d*_*in*_, average duration of untreated infection. The model represents a situation where disease is being controlled using treatment of symptomatic/clinical malaria. Uninfected individuals become infected at a rate proportional to the overall prevalence of malaria infection. Recovery takes place either as treatment of clinical malaria at a given coverage or as natural clearance of the parasites. Immunity is assumed to be lost if immune uninfected individuals are not challenged for a given time period. The model equations and a detailed description are found elsewhere.^4^ The model was used to demonstrate the potential for combinations of interventions for elimination programmes. This model does not include explicit vector population and transmission dynamics, multiple levels of immunity, or the liver or asexual stages of infection. It is a simple representation of an extremely complex biological system and is for the purposes of understanding the more general behaviour of malaria transmission specifically during elimination and could be used as a first step by policy-makers for strategy planning for a few years ahead.

Policy-makers have tended not to include mathematical modelling in their planning of national and regional malaria control strategies.^2^ In its place there has been a reliance on malaria surveillance to estimate the impact of control programmes. As illustrated in Figure 1, this is dangerous when the target is elimination as reliance on surveillance data alone can result in false reassurance that elimination has been successful. Surveillance data can also often be very unreliable and poor data quality can give misleading results. This diminishes its value for policy-makers and in this context modelling can be particularly helpful as an additional source of guidance. The potential contribution of modelling to predict timelines for malaria elimination has been presented elsewhere.^15^

To overcome policy-maker's distrust of modelling, there is a need for it to be more pragmatic and intervention-focused.^2^, ^8^ Country- or region-specific modelling is more practically useful to control programmes than general conclusions as it takes account of factors that vary between areas, e.g. bed nets are much less effective where mosquitoes bite during the day and patterns of drug and insecticide resistance are highly variable. Modelling can also prevent unnecessary interventions that would waste money and can help determine the best use of resources. Ideally, to optimise their impact on policy, malaria elimination models need to be developed alongside control programmes. They can be used in the initial stages to help plan which interventions to employ and then refined as surveillance data are collected. More realistic, useful and situation-specific recommendations can then be made. A major limiting factor preventing full integration of modelling in malaria control activities has been the lack of availability of location-specific surveillance data of sufficient quality and timeliness to inform ongoing model development in real-time.

Integration of modelling into control programmes has occurred for other diseases where the interplay between modelling and policy is far more developed, e.g. onchocerciasis^2^ and vaccination programmes.^16^ The UK Health Protection Agency regularly employs modelling to help plan major infectious disease control programmes, e.g. influenza.^17^, ^18^ Although there continue to be great strides in the mathematical modelling of malaria with a number of theoretical models providing useful insights,^19^, ^20^ it is only very recently that the role of pragmatic, prospective, intervention-focused modelling to answer specific disease control questions^2^ has begun to be recognised as important in malaria.^3^, ^21^, ^22^, ^23^, ^24^, ^25^ There remains a notable lack of models that specifically compare different modalities for malaria control or that examine the impact of combinations of interventions.^3^, ^4^, ^12^, ^26^, ^27^ Perhaps most surprising of all is that despite the recent upsurge in interest in eradication and elimination there are few models that specifically consider these.^3^, ^4^, ^19^, ^21^, ^23^, ^26^, ^27^ Many models of malaria control are not appropriate for examining elimination and some of the reasons for this will be discussed in the next section.

### 2.2 Modelling methods for elimination

**Figure 2:**
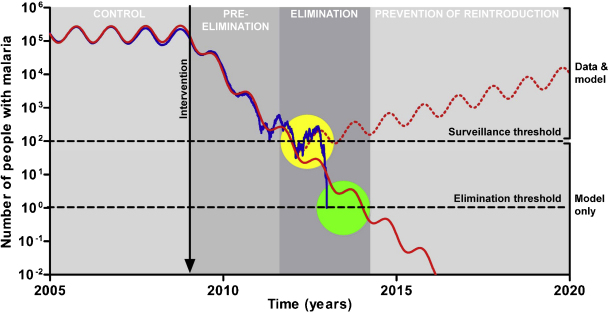
Deterministic (red line) versus stochastic (blue line) modelling of malaria elimination using a model previously published elsewhere.^3^ Only one run of the stochastic model is shown for clarity. The phases of the WHO malaria control-to-elimination continuum1 are indicated by the shaded background. The ‘limit of detection by surveillance’ indicates the number of cases below which a malaria surveillance programme is unlikely to detect any malaria, thus suggesting ‘apparent elimination’ (yellow circle). An arbitrary example is shown in the figure. Because of this detection limit, only the upper portion of the figure can be represented by surveillance data (‘data & model’), whereas the lower portion can only be represented by modelling predictions (‘model only’). Improving the sensitivity of surveillance would lower this detection limit. For ‘true elimination’ to occur (green circle), the number of malaria cases must fall below the ‘elimination threshold’ (<1 case). Only a perfect surveillance system detecting every case would have a limit of detection by surveillance equal to the elimination threshold. This is not generally the case in the field where surveillance systems are far from perfect and can miss many cases. Thus, the limit of detection by surveillance is generally above the elimination threshold. If malaria control interventions are stopped inappropriately early when apparent elimination occurs (red dotted line), numbers of cases begin to increase again. Modelling gives an indication of how long control measures would need to be continued to achieve true elimination. (For interpretation of the references to color in this figure legend, the reader is referred to the web version of the article.)

Infectious disease elimination is a unique situation that requires specific considerations in modelling. The most frequently employed modelling technique to investigate infectious disease dynamics is deterministic modelling. This is the most efficient method for considering multiple scenarios and producing initial results and recommendations quickly, as the model development and run times are very short.^4^ It is also most appropriate when data are sparse. An example of such a deterministic model developed for malaria elimination is shown in Figure 1.^4^ However, this style of modelling is not appropriate for the end stages of elimination strategies where numbers of infected individuals become very low and individual variation in risk, movement and treatment-seeking behaviour become more significant. Deterministic modelling relies on the assumption that the population under consideration is large. As illustrated in Figure 2, when numbers of infected cases are small, this can lead to unrealistic persistence of infection in a population beyond when it would have been eliminated. Stochastic models consider whole numbers of individuals and include a degree of random variation that more accurately reproduces behaviours in nature. They are run multiple times, each giving slightly different outputs, and can thus give probabilities of particular outcomes as results rather than the exact numbers that come from deterministic models. This can give an indication of the degree of risk of a particular strategy not succeeding. However, stochastic models are more complex and time consuming to develop, require more data and take much longer to run.

Another important consideration when modelling elimination is the common simplifying assumption of homogeneous transmission for the entire population. Although this allows for simpler models (e.g. Figure 1), it can lead to an overestimation of the impact of control measures and unrealistic optimism about the ease of elimination.^15^ In reality, malaria transmission can be highly heterogeneous. Failure of an elimination attempt is likely to result from ongoing transmission in spatial reservoirs. To capture this heterogeneity, a transmission dynamic spatial model is required, that is a model that includes multiple interacting populations (or individuals) in geographically defined areas, each with their own characteristics. This type of model also allows the investigation of spatially explicit control measures, e.g. selectively distributing bed nets to high transmission foci. Such strategies can greatly increase the impact and cost effectiveness of control measures.^28^ Spatial models allow inclusion of population migration between and within countries, which can be an important contributor to the spread of malaria, and allows for exploration of country versus regional approaches to elimination. To parameterise realistic spatial models correctly requires much more data than their non-spatial equivalent. In data-poor situations, the extra effort required to produce a spatial model may give little useful extra information. They also require more computing power and this increases as the number of metapopulations increases. Partly because of these difficulties, little transmission dynamic spatial modelling of malaria has been attempted previously and there have been no substantial attempts to model migration patterns of malaria infections.^29^ Rather, there is a substantial body of empirical spatial statistical models that incorporate spatial autocorrelation structure into regression models to produce static geographical patterns of infection, e.g. spatial mapping of malaria prevalence from the Malaria Atlas Project, which is being used to help with planning spatially explicit control measures in Kenya.^30^ As such statistical models ignore transmission dynamics, they cannot be used to predict the impact of malaria elimination measures.

There are several models of malaria elimination in development as part of control programmes, although few details of these have yet been published. A prominent consortium of modellers have agreed that multiple models with different approaches and assumptions to answer specific questions are necessary for malaria elimination.^6^ A combination of simple and complex models is probably ideal.^4^ Following this paradigm, the initial approach for the malaria elimination effort in Western Cambodia includes deterministic and stochastic models with a spatial framework currently under development. This is described in detail elsewhere.^3^

No matter how informative a model is about the relative impact of different strategies, ultimately the choice of strategy for use on the ground will likely be heavily influenced by cost and affordability. Highly effective strategies for malaria elimination may be economically inefficient. Economics therefore must be included in the modelling to identify the most efficient mix of interventions. The potential role of modelling in guiding the economics of malaria elimination and what will be required for this to provide useful results will be explored in the next section.

## 3 Guiding the economics

An area of great potential benefit in planning malaria elimination is using modelling to predict which strategies are likely to be the most cost efficient. This can be quantified by weighing the overall economic costs against the economic benefits (cost–benefit analysis), the health benefits (outcome measures such as cost per quality-adjusted life-year, cost–utility analysis) or cost per malaria case averted (cost-effectiveness analyses).

### 3.1 Costs

Previous economic analyses of malaria elimination programmes have been relatively simple and focused mainly on costing interventions and much less on the benefits. At the beginning of the GMEP in 1955 there were very little data available on the costs and benefits of malaria control. Over the succeeding years of the GMEP, some simple cost–benefit analyses were carried out to evaluate national elimination and control programmes. These produced widely varying cost–benefit ratios and probably underestimated the benefits through concentrating mainly on estimated effects on labour supply and failing to encompass adequately the value of health gains.^31^ These analyses were largely hypothetical and prospective and were not later tested against new data.^31^ Since then there have been some attempts to quantify the economic burden of malaria^32^ whilst mostly ignoring the financial costs of control.^31^ There has also been work to compare the costs of long-term control with those of elimination, with differing results.^31^ More recently, the Gates Foundation, together with Roll Back Malaria, published combined cost estimates for global malaria control and elimination of US$6 billion annually, falling to US$1.5 billion over around three decades.^33^ However, the additional costs of elimination or eradication over control were not given. These and other previous costing exercises have produced a very broad spread of answers. Reasons for this disagreement include the extensive uncertainty surrounding the likely timescale of eradication, the presumed appearance of new more effective technologies with unknown cost, and wide variation between countries with different estimated target coverages and costing approaches.^31^ The costs of particular interventions are highly variable between different locations depending on, for example, the established health system, disease surveillance and other infrastructure (is the intervention to be vertical or horizontal?) and locally negotiated contracts. It is particularly difficult to quantify the cost of strengthening a health system if this is required for an intervention to be successfully implemented, and it is not always clear to what extent such costs should be considered part of programme expenditure.

### 3.2 Benefits

Quantifying the benefits of malaria elimination is a tremendous challenge. There are different types of benefits, some obvious and some less so: economic and health-related direct benefits; benefits to individuals and more nebulous indirect benefits to the wider community; and immediate versus attributable longer term benefits. Malaria occurs predominantly in countries with very limited resources where even the direct economic benefits can be difficult to quantify. Numbers of malaria cases in many countries are woefully inaccurate.^34^ Many disease surveillance systems are inadequate, with numerous misdiagnoses, and patients are frequently not included in government statistics as they seek cheaper and/or more convenient private sector alternatives or avoid formal healthcare altogether. Even if the direct healthcare-associated costs and consequences of treating a single case of malaria can be quantified, preventing one episode of malaria has much wider effects. There is an indirect impact on the individual's families and communities, including avoiding lost income and the costs of caring for that individual. There is a reduction in onward malaria transmission, preventing further illnesses and associated costs in the wider community and economy.^32^ Economic modelling has traditionally ignored these transmission dynamics and only considered direct costs and benefits. It is also difficult to know how much of these future savings are a direct result of the intervention, as the further into the future one looks the more likely there has been some other confounding change, e.g. long-term economic development. To deal with this, economic evaluations often ‘discount’ the value of future costs and benefits to estimate their present day value. This reduces the predicted future costs and benefits of an intervention by a fixed percentage each year to reflect the lesser value placed on future compared with immediate costs and benefits.^31^ The choice of discounting rate can be very influential. When comparing the costs of elimination with those of ongoing control, for instance, it has been shown that higher discount rates favour control strategies, whilst low values or no discounting result in elimination strategies being the preferred option.^35^ Similarly, when discounting future costs and benefits, the time horizon of the analysis and the estimates for when, for instance, elimination is to be achieved will also play a critical role in determining its benefits in present day value. Longer durations to elimination imply a lesser current value for its benefits, which could once again suggest that control strategies be preferred, particularly if the costs of elimination strategies are assumed to be incurred in the much nearer future.

### 3.3 Combining disciplines

To date, most health economic modelling of infectious diseases has used static considerations of fixed disease burdens. This is acceptable for malaria control where disease burden and transmission remain constant. However, during elimination programmes the disease burden and transmission intensity of malaria change dynamically and static models are inappropriate. Although Markov decision analytic models have been used for this, it is more realistic to use a population dynamic transmission model.^36^ At minimum, therefore, static, decision tree-based evaluations could be substituted by use of Markov models to capture time-dependent transition probabilities for changes in factors such as transmission intensity and the development of drug resistance. This is particularly important where these factors are influenced by the implementation of the interventions being evaluated (e.g. widespread use of ACTs and rapid diagnostic tests). In this case these parameters are exogenous to the economic models and can be derived from independent population dynamic transmission models. Alternatively, population dynamic models focusing on different strategies towards elimination could themselves be expanded to include cost parameters endogenously, providing cost-effective evidence for their implementation. Incorporation of realistic population dynamic transmission modelling with economic modelling has rarely been attempted for any infectious disease, some notable exceptions being multidrug-resistant tuberculosis (TB),^37^ TB in high HIV prevalence settings^38^ and some vaccination programmes,^16^ e.g. pandemic influenza^39^ and human papillomavirus.^40^ For all of these examples, the modelling provided important insights and was used to determine the most cost effective of a range of interventions to control the particular infectious disease. To do this adequately requires quite sophisticated costings and assessment of benefits and thus collaboration between transmission dynamic and economic modellers is needed. There is, however, a pay-off to consider between complexity of the transmission model and the economic model, as if both are highly complex the combination may become unwieldy. A compromise must thus be reached.

Given these limitations, it is important that the economic analysis is relevant to the policy-makers it aims to inform. For a national malaria programme, the broader benefits to the economy associated with malaria elimination are of lesser interest than a detailed assessment of the precise costs of different elimination strategies. Similarly, they are unlikely to be concerned with alternative uses of resources other than those aimed at controlling or eliminating malaria. In other contexts, it might be essential to model the broader costs and consequences of malaria elimination, where for instance a government might have to decide between investing in an elimination programme or in other health or non-health interventions.

### 3.4 Pre-elimination stage challenges

Another important role of modelling economics in malaria elimination is in helping to persuade individuals and policy-makers to sustain interventions once the malaria burden falls below the limit of detection of surveillance (Figure 1). When there appears to be little or no disease, the public and politicians may lose interest in malaria elimination measures thinking them to be no longer necessary.^31^ This can affect their acceptability, e.g. people may be less willing to accept MDA or spraying of insecticide in their dwelling. There is a big challenge here in communicating the need for ongoing efforts to ensure high coverage of interventions in order to achieve true elimination and this will require engagement at all levels.^31^ It is important to predict how long an intervention needs to be sustained in order to achieve true elimination in an area,^15^ and economic modelling can predict how much this is likely to cost. This is likely to be a significant proportion of the malaria elimination campaign's budget (it will be more the less sensitive the surveillance system is). The WHO define the final stage of malaria elimination as ‘prevention of reintroduction’.^1^ This requires continued investment in control activities for a minimum of 3 years after elimination is thought to have been achieved. This investment will need to be particularly large in areas where there is population migration of infected cases from other areas, and the required period of 3 years for WHO certification may be insufficient. Modelling can go a long way to informing these planning processes. Quantifying the relative cost of stopping a malaria elimination effort too early would provide a very important message.

Current long-term plans for malaria elimination are global. Ultimately, the WHO wants to eradicate malaria. There are a number of additional considerations here. The most useful transmission dynamic and economic modelling is that which is supported by the best data. In many areas of the world, the required data are sparse or non-existent. It is important to establish how and to what degree conclusions from modelling and cost–benefit analysis can be extended from settings with good data to areas with poor data. The Malaria Atlas Project has collated malaria prevalence data from sites across the globe and has produced a map of malaria prevalence that uses these data and interpolates estimated prevalences for areas where there are no data.^41^ A great strength of this project is that it includes a map indicating the degree of uncertainty of these estimates. Such a quantification of uncertainty would need to be developed when extrapolating economic analyses to data-poor areas. A full economic evaluation should consider not only which policy is likely to be the best, but should fully quantify uncertainty associated with policy decisions.

## 4 Conclusions

If we are to aim for elimination and eradication as goals for malaria control worldwide^1^, ^33^ then we need to learn from the mistakes and successes of the mid 20th century GMEP. There are many more powerful strategies available to us today and it is imperative that we have some way of identifying which are most likely to be successful in different epidemiological settings. Transmission dynamic mathematical modelling is essential to this. Whichever interventions are chosen need to be the most cost efficient possible. Enormous financial resources are being devoted to malaria control, vastly more than ever before, but funding is not unlimited. Successful elimination in some areas should help reassure donors that investing in malaria is worthwhile and ensure that funding is sustained. Economic modelling will be an invaluable tool to aid in this prioritising process. The combined tool of transmission dynamic mathematical modelling plus sophisticated economic analysis will only be as good as the data on which it is based. As interventions are rolled out it is imperative that high-quality surveillance data are collected, collated and made widely available. The models will improve and increase in precision as a result. If malaria elimination and eradication can be shown to be feasible at a reasonable cost, this would be powerful motivation for policy-makers to remove the barriers to their implementation.^31^

## Authors’ contributions

All authors were involved in conceptualising, drafting and revising the review; RJM, YL and BSC carried out the literature review. LJW is guarantor of the paper.

## Funding

The Mahidol–Oxford Tropical Medicine Research Unit is funded by the Wellcome Trust of Great Britain. RJM is funded by a Fellowship from the British Infection Society. RJM, WP, SY and LJW also receive funding from the Bill & Melinda Gates Foundation.

## Conflicts of interest

None declared.

## Ethical approval

Not required.
